# Machine Learning Prediction Models for Postoperative Stroke in Elderly Patients: Analyses of the MIMIC Database

**DOI:** 10.3389/fnagi.2022.897611

**Published:** 2022-07-18

**Authors:** Xiao Zhang, Ningbo Fei, Xinxin Zhang, Qun Wang, Zongping Fang

**Affiliations:** ^1^Department of Anesthesiology and Perioperative Medicine, Xijing Hospital, Fourth Military Medical University, Xi’an, China; ^2^Department of Orthopedics and Traumatology, The Duchess of Kent Children’s Hospital at Sandy Bay, The University of Hong Kong, Hong Kong, Hong Kong SAR, China

**Keywords:** stroke, machine learning, prediction model, post-operative, MIMIC database

## Abstract

**Objective:**

With the aging of populations and the high prevalence of stroke, postoperative stroke has become a growing concern. This study aimed to establish a prediction model and assess the risk factors for stroke in elderly patients during the postoperative period.

**Methods:**

ML (Machine learning) prediction models were applied to elderly patients from the MIMIC (Medical Information Mart for Intensive Care)-III and MIMIC-VI databases. The SMOTENC (synthetic minority oversampling technique for nominal and continuous data) balancing technique and iterative SVD (Singular Value Decomposition) data imputation method were used to address the problem of category imbalance and missing values, respectively. We analyzed the possible predictive factors of stroke in elderly patients using seven modeling approaches to train the model. The diagnostic value of the model derived from machine learning was evaluated by the ROC curve (receiver operating characteristic curve).

**Results:**

We analyzed 7,128 and 661 patients from MIMIC-VI and MIMIC-III, respectively. The XGB (extreme gradient boosting) model got the highest AUC (area under the curve) of 0.78 (0.75–0.81), making it better than the other six models, Besides, we found that XGB model with databalancing was better than that without data balancing. Based on this prediction model, we found hypertension, cancer, congestive heart failure, chronic pulmonary disease and peripheral vascular disease were the top five predictors. Furthermore, we demonstrated that hypertension predicted postoperative stroke is much more valuable.

**Conclusion:**

Stroke in elderly patients during the postoperative period can be reliably predicted. We proved XGB model is a reliable predictive model, and the history of hypertension should be weighted more heavily than the results of laboratory tests to prevent postoperative stroke in elderly patients regardless of gender.

## Introduction

Stroke, also called cerebrovascular accident, includes the neurological pathology of the brain arteries that can result from ischemia or hemorrhage (Boursin et al., 2018). Stoke ranks as the second-leading cause of mortality and disability worldwide behind ischemic heart disease and thereby become a major health-related challenge (Merino, 2014; Sirsat et al., 2020). Stroke also gives approximately 16,000,000 individuals worldwide various motor and cognitive impairments, which are often unavoidable sequelae in stroke patients. These sequelae greatly aggravate the social and family burden (Di Carlo, 2009). People with advanced age, surgery patients and ICU patients are at high risk of stroke (Mantz et al., 2010; Biteker et al., 2014; Dong et al., 2017). Consequently, it’s urgent to establish an advanced model that can help to predict and diagnose stroke. The early correct detection of stroke will lay a solid foundation to efficiently prevent and treat stroke and will greatly improve the prognosis of surgery. A prediction model is a practicable way to achieve the above goals and several attempts have been made (Maravic-Stojkovic et al., 2014; Khattar et al., 2016; Dunham et al., 2017; Sporns et al., 2017; Zhou et al., 2020). However, there is still a demand for models that can predict stroke in elderly patients after surgery.

Machine Learning (ML), as a mature and scientific modeling method, is attracting more attention than traditional modeling approaches such as the Cox proportional hazard model. ML is a pivotal part of artificial intelligence (AI), it can achieve self-optimization by learning complex structure with numerous variables and data (Bi et al., 2019). So far, ML has wide application in several fields, including search engines, sales and marketing, and autonomous driving (Deo, 2015; Jiang et al., 2017; Handelman et al., 2018; Connor, 2019), as well as medical diagnostics and clinical research (Heo et al., 2019; Saber et al., 2019; Sirsat et al., 2020). During the past few decades, several studies were conducted on the improvement of stroke diagnosis using ML, most of them obtained satisfying results, which would be of great value in early prognosis of stroke (Asadi et al., 2016; Cox et al., 2016; Bacchi et al., 2020; Wu and Fang, 2020). For example, the electromyography (EMG) based muscular activity monitoring system, electroencephalography (EEG) based neuronal firing activity monitoring system and electrocardiogram (ECG) based monitoring system have been applied into the early identification and prognosis of stroke, which are also beneficial to post-stroke rehabilitation (Robinson et al., 2003; Hussain and Park, 2020, 2021a,b).

We obtained our data from two public clinical databases, which contains rich and complete clinical data. In the practice of machine learning modeling, we utilized not only subjects from MIMIC-VI for internal validation but also samples from the MIMIC-III database for further external testing. The goal of the present study is to introduce a prediction model for postoperative stroke in elderly patients. We applied seven machine learning method in this research combined with iterative SVD data imputation and SMOTENC method, which would deliver an accurate and quick prediction outcome. Based on our results, the perioperative patients with high risk of stroke could be found and treated as early as possible, which would shed new light on the prevention of stroke.

## Materials and Methods

### Database and Study Design

We obtained our data from two publicly available retrospective multigranular clinical databases, MIMIC-III and MIMIC-VI, which are high-quality database of admitted patients from 2000 to 2014 and from 2014 to 2018, respectively. They have large samples with comprehensive clinical information. The 80% percent of the samples from MIMIC-VI, chosen randomly, were regarded as the development set, and the remaining 20% were regarded as the validation set. Besides, the samples from MIMIC-III were applied as an independent testing set to further evaluate the applicability of the established models and predictors.

### Subjects and Outcomes

**FIGURE 1:**
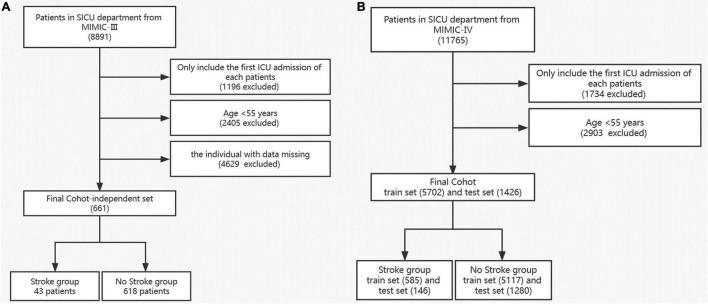
Flow diagram of the selection process of patients in MIMIC III **(A)** and MIMIC VI **(B)**.

In this study, subjects who were admitted to the SICU (surgery intensive care unit) with age > 55 years were selected. All these patients should include vital signs, complications and laboratory results. As shown in Figure 1, subjects younger than 55 years were excluded. Missing values of enrolled individuals in MIMIC-VI were filled with the iterative SVD data imputation method. Only patients with complete data in MIMIC-III were kept. We finally screened 661 patients from MIMIC-III and 7,128 patients from MIMIC-VI into the study. Incidence of stroke was used as the outcome measure. Then we separated patients into the stroke group and non-stroke group based on their diagnosis in the hospital.

We select predictors according to what features chosen in the previous research (Heo et al., 2019; Sirsat et al., 2020; Wu and Fang, 2020), as well as our clinical experience. Predictors with missing data more than 30% in MIMIC-III and MIMIC-VI, such as bicarbonate, were excluded. The predictors included (a) demographic information: age, sex, ethnicity and BMI index; (b) comorbidities: peripheral vascular disease, hypertension, chronic pulmonary disease, diabetes, renal disease, liver disease, peptic ulcer disease, sepsis, congestive heart failure, cancer, and rheumatic disease; (c) the first-day laboratory results in the ICU: the mean level of glucose; the lowest and mean levels of Spo_2_, the lowest and highest levels of anion gap, albumin, bilirubin total, creatinine, hematocrit, hemoglobin, WBC (white blood cells), lactate, platelets, potassium, PTT (partial thromboplastin time), PT (prothrombin time), INR (international normalized ratio), and BUN (blood urea nitrogen); and (e) the first-day vital signs in the ICU: the highest and mean levels of heart rate, SBP (systolic blood pressure), DBP (diastolic blood pressure), and MBP (mean blood pressure) (Dunham et al., 2017; Wu and Fang, 2020; Bolourani et al., 2021).

**FIGURE 2:**
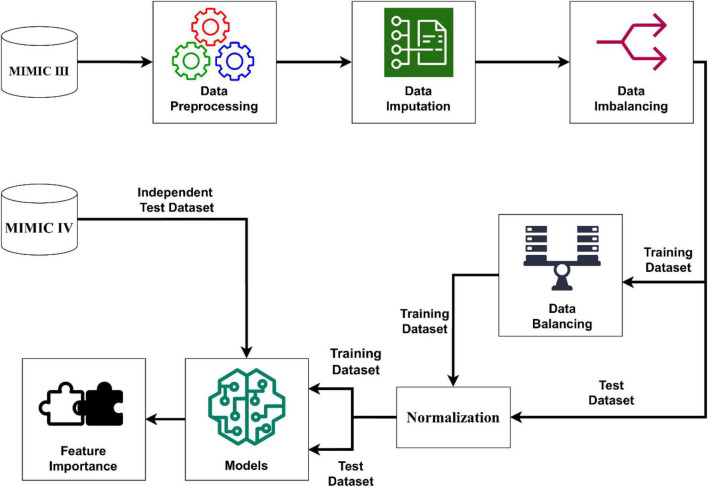
Schematic illustration to the performance of the stroke prediction model. The SMOTENC balancing technique was applied to training dataset before establishing the model due to the imbalanced ratio of non-stroke to stroke patients in this work. After the normalization of data was completed, we applied seven machine learning methods to train and test models with the training dataset, test dataset and the independent test dataset. Finally, we can get model-based importance of features.

We extracted the target subjects with all of the above information and outcome measures *via* navicat premium12 software. Data cleaning was completed by Stata software after the data extraction. Firstly, individuals who met the exclusion criteria were excluded. Secondly, the extreme values and outliers were deleted. For data in MIMIC- VI, we excluded subjects with missing values accounting for more than 5% of the predictive features. Imputation method was used to handle with the missing values. For data in MIMIC- III, we merely keep variables with complete values, which was treated as an independent validation set. Therefore, the subsets were established for the final analyses. The process of establishing models was well illustrated in Figure 2.

### Statistical Analysis

We compared the characteristics above between the stroke group and the non-stroke group, also between the development cohort and validation cohort. Differences in normally distributed data are described as mean ± *SD* (standard deviation) and were compared by the Students’ *t*-test, while differences in non-normal data are described as median and IQR (interquartile range) and were compared by a non-parametric test. Differences in rate and constituent ratio data are presented as numbers and percentages, and they were compared with the chi-squared test or a non-parametric test.

The missing values of the training set and the verification set in the MIMIC-VI database were reasonably filled in with the iterative SVD data imputation method (Troyanskaya et al., 2001; Di Lena et al., 2019). The results of previous literature suggested that machine learning methods with data balancing methods had better performance in stroke prediction compared with imbalanced data (Wu and Fang, 2020). And in this research, a variant of SMOTE called SMOTE-NC is applied in this research to solve the problem of category imbalance because of the categorical features. All classifiers are trained with an equal number of training samples per class through oversampling (Pears et al., 2014; Bolourani et al., 2021).

In this article, several kinds of classifiers are used in machine learning methods to classify strokes. We used a Python library called Scikit-learn to build our classifier. This Python package provides several classification algorithms and is a powerful and useful open-source machine learning toolkit. The details of performing the stroke prediction model are shown in Figure 2. We employed 7 machine learning algorithms including KNN (k-nearest neighbor), SVM (support vector machine), MLP (multilayer perceptron), LR (logistic regression), DT (decision tree), RF (random forest) and XGBoost (extreme gradient boosting) to establish a stroke prediction model with the training set. The hyperparameters which we used in 7 machine learning algorithms came from default setting in scikit-learn package. Eg: The hyperparameter of KNN is k and the default setting in scikit-learn is “k = 3,” which was used in this study. The performance of the models was weighed by the AUROC (area under the receiver operating characteristic curves) (Zhou et al., 2020). For the best-performing model, the importance of the predictors was evaluated and computed with the information gain. SAS 9.4, R software 3.6.1, and MATLAB 9.9 were used for statistical analyses.

## Results

### Patient Characteristics

With the data obtained from the common database, we finally identified 7,128 and 661 patients from MIMIC-VI and MIMIC-III, respectively. The screening process is shown in Figure 1. Predictors with too much missing data, such as bicarbonate, were excluded. In the current research, we ultimately included 51 predictors. The age, vital signs and partial laboratory results are shown as mean and *SD*; other laboratory results are shown as median and IQR. Sex, race, and comorbidities are shown as number and percentage. Patients in the MIMIC-VI database were divided into stroke group (*n* = 731) and non-stroke group (*n* = 6,397). Their baseline characteristics are shown in Table 1. The stroke group subjects were older (74.0 ± 10.5 vs. 72.1 ± 10.4) and had a higher incidence of hypertension. Additionally, both the stroke group and the non-stroke group were similar in BMI and sex distribution. Patients in the MIMIC-VI database were randomly separated into training and validation sets at a ratio of 8:2, while the MIMIC-III database made up the independent testing set. Patients with stroke in the training set, validation set and independent testing set accounted for 10.3, 10.2, and 6.51%, respectively. The training cohort included 5,117 non-stroke subjects and 585 stroke subjects. The validation cohort had 1,280 non-stroke subjects and 146 stroke subjects. The independent testing cohort had 618 non-stroke subjects and 43 stroke subjects. Patients in the training and validation cohorts were similar in demographic characteristics, the incidence of various comorbidities, laboratory results and vital signs, as shown in Supplementary Table 1. For the independent testing set, constituted by data from MIMIC-VI database, the population included was quite different, and only patients with complete values were included, which is a requirement of an independent testing cohort. The characteristics of patients in the independent testing set were shown in Supplementary Table 2.

**TABLE 1:** Characteristics of stroke and non-stroke patients in the training and validation sets of the MIMIC-IV database.

Variables	Total (*n* = 7,128)	Non-stroke (*n* = 6,397)	Stroke (*n* = 731)	*P*
**Demographic characteristics**				
Age, mean ± *SD*	72.3 ± 10.4	72.1 ± 10.4	74.0 ± 10.5	<0.001
Gender, female *n* (%)	3,426 (48.1)	3,082 (48.2)	344 (47.1)	0.584
**Race, *n* (%)**				0.039
Asian, *n* (%)	222 (3.1)	193 (3)	29 (4)	0.198
Black, *n* (%)	592 (8.3)	538 (8.4)	54 (7.4)	0.38
White, *n* (%)	5,083 (71.3)	4,584 (71.7)	499 (68.3)	0.06
Other, *n* (%)	1,231 (17.3)	1,082 (16.9)	149 (20.4)	0.022
BMI, mean ± *SD*	1300.8 ± 85503.1	1404.9 ± 90219.6	389.4 ± 7628.6	0.761
**Comorbidities**				
CHF, *n* (%)	1,483 (20.8)	1,399 (21.9)	84 (11.5)	<0.001
PVD, *n* (%)	927 (13.0)	858 (13.4)	69 (9.4)	0.003
Hypertension, *n* (%)	2,794 (39.2)	2,278 (35.6)	516 (70.6)	<0.001
CPD, *n* (%)	1,712 (24.0)	1,595 (24.9)	117 (16)	<0.001
Diabetes, *n* (%)	2,070 (29.0)	1,879 (29.4)	191 (26.1)	0.074
Renal_disease, *n* (%)	1,349 (18.9)	1,267 (19.8)	82 (11.2)	<0.001
Liver_disease, *n* (%)	844 (11.8)	817 (12.8)	27 (3.7)	<0.001
PUD, *n* (%)	204 (2.9)	201 (3.1)	3 (0.4)	<0.001
Cancer, *n* (%)	1,405 (19.7)	1,322 (20.7)	83 (11.4)	<0.001
Rheumatic_disease, *n* (%)	259 (3.6)	245 (3.8)	14 (1.9)	0.012
Sepsis, *n* (%)	3,033 (42.6)	2,773 (43.3)	260 (35.6)	<0.001
**Laboratory results**				
Spo2_min, mean ± *SD*	91.8 ± 7.1	91.8 ± 6.7	92.1 ± 9.6	0.383
Spo2_mean, mean ± *SD*	96.8 ± 2.6	96.8 ± 2.5	97.0 ± 3.2	0.14
Aniongap_min, mean ± *SD*	13.5 ± 3.3	13.4 ± 3.4	13.8 ± 2.6	0.004
Aniongap_max, mean ± *SD*	16.2 ± 4.3	16.3 ± 4.4	16.0 ± 3.1	0.182
Albumin_min, mean ± *SD*	3.3 ± 0.5	3.3 ± 0.5	3.6 ± 0.4	<0.001
Albumin_max, mean ± *SD*	3.4 ± 0.5	3.4 ± 0.5	3.7 ± 0.4	<0.001
Glucose_mean, mean ± *SD*	140.5 ± 49.0	140.6 ± 48.5	139.8 ± 53.5	0.679
Potassium_min, mean ± *SD*	3.8 ± 0.4	3.8 ± 0.4	3.7 ± 0.3	<0.001
Potassium_max, mean ± *SD*	4.1 ± 0.5	4.1 ± 0.5	3.9 ± 0.4	<0.001
Bilirubin_total_min, median (IQR)	0.8 (0.4, 1.6)	0.9 (0.4, 1.6)	0.8 (0.4, 1.2)	<0.001
Bilirubin_total_max, median (IQR)	1.0 (0.4, 1.9)	1.0 (0.4, 2.0)	0.8 (0.4, 1.4)	<0.001
Creatinine_min, median (IQR)	0.9 (0.7, 1.2)	0.9 (0.7, 1.2)	0.8 (0.7, 1.1)	0.002
Creatinine_max, median (IQR)	1.0 (0.8, 1.4)	1.0 (0.8, 1.5)	0.9 (0.8, 1.2)	<0.001
Lactate_min, median (IQR)	1.5 (1.2, 1.9)	1.5 (1.2, 1.9)	1.4 (1.2, 1.7)	<0.001
Lactate_max, median (IQR)	2.0 (1.4, 2.9)	2.1 (1.4, 2.9)	1.9 (1.4, 2.4)	<0.001
Platelets_min, median (IQR)	193.0 (141.0, 253.0)	192.0 (139.0, 254.0)	196.0 (157.0, 246.5)	0.12
Platelets_max, median (IQR)	218.0 (165.0, 285.0)	218.0 (163.0, 287.0)	216.0 (171.0, 273.0)	0.816
Ptt_min, median (IQR)	28.3 (25.0, 32.6)	28.4 (25.2, 33.1)	26.8 (23.6, 30.0)	<0.001
Ptt_max, median (IQR)	30.5 (26.5, 40.2)	30.8 (26.7, 42.0)	28.6 (24.9, 33.3)	<0.001
Inr_min, median (IQR)	1.2 (1.1, 1.4)	1.2 (1.1, 1.4)	1.1 (1.0, 1.2)	<0.001
Inr_max, median (IQR)	1.2 (1.1, 1.6)	1.2 (1.1, 1.7)	1.1 (1.1, 1.4)	<0.001
Pt_min, median (IQR)	13.0 (11.7, 15.1)	13.1 (11.7, 15.3)	12.3 (11.4, 13.6)	<0.001
Pt_max, median (IQR)	13.8 (12.1, 18.8)	13.9 (12.1, 19.3)	13.0 (11.8, 15.5)	<0.001
Bun_min, median (IQR)	18.0 (12.0, 26.2)	18.0 (13.0, 27.0)	16.0 (12.0, 21.0)	<0.001
Bun_max, median (IQR)	20.0 (15.0, 31.0)	21.0 (15.0, 32.0)	19.0 (15.0, 24.0)	<0.001
Wbc_min, median (IQR)	9.3 (6.8, 12.6)	9.3 (6.8, 12.7)	9.2 (7.1, 11.9)	0.951
Wbc_max, median (IQR)	11.7 (8.6, 15.9)	11.8 (8.6, 16.1)	11.1 (8.7, 14.5)	<0.001
**Vital signs**				
TP_mean, mean ± *SD*	100.8 ± 20.3	101.3 ± 20.5	96.9 ± 18.4	<0.001
HR_max, mean ± *SD*	82.4 ± 15.0	82.8 ± 15.2	78.5 ± 12.8	<0.001
HR_mean, mean ± *SD*	153.9 ± 23.3	152.8 ± 23.3	164.2 ± 21.2	<0.001
Sbp_max, mean ± *SD*	123.5 ± 17.4	122.5 ± 17.4	132.3 ± 14.4	<0.001
Sbp_mean, mean ± *SD*	88.0 ± 20.2	87.7 ± 20.2	90.9 ± 19.4	<0.001
Dbp_max, mean ± *SD*	62.6 ± 10.7	62.3 ± 10.8	64.8 ± 10.1	<0.001
Dbp_mean, mean ± *SD*	106.6 ± 24.0	106.1 ± 24.1	110.6 ± 22.5	<0.001
Mbp_max, mean ± *SD*	79.5 ± 11.0	79.1 ± 11.1	83.0 ± 9.8	<0.001
Mbp_mean, mean ± *SD*	36.9 ± 0.6	36.8 ± 0.6	36.9 ± 0.6	<0.001

*Continuous variables are presented as the median and interquartile range (IQR). Count data are presented as numbers and percentages. Severe respiratory failure, severe coagulation failure, severe liver failure, severe cardiovascular failure, severe central nervous failure, and severe renal failure refer to the scores of the specific organ or system that scored 4 in the SOFA scheme. The definition of the medical condition was based on the ICD-9 code. A mean, minimum, or maximum parameter refers to the mean, the highest, or the lowest level of the parameter on the first day of ICU admission. CHF, congestive heart failure; PVD, peripheral vascular disease; CPD, chronic pulmonary disease; PUD, chronic pulmonary disease; Spo2, finger pulse oxygen saturation; ptt, partial thromboplastin time; INR, international normalized ratio; pt, prothrombin time; BUN, blood urea nitrogen; wbc, white blood cells; TP, temperature; HR, heart rate; sbp, systolic blood pressure; dbp, diastolic blood pressure; mbp, mean blood pressure.*

### Prediction Models

**FIGURE 3:**
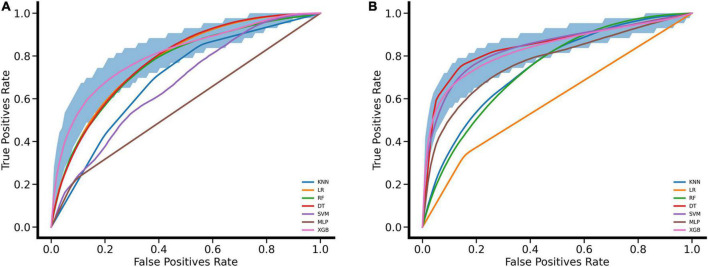
Performance evaluation for seven machine learning algorithms with ROC curves. **(A)** ROC curves were drawn for the validation set based on MIMIC VI performed by leaving 20% as a testing set and using the rest for the training set. **(B)** ROC curves were drawn for the independent testing set based on MIMIC III. The mean ROC curve of XGB is shown in pink and its corresponding 95% confidence interval is shown in deep blue.

The process of performing the stroke prediction model was illustrated in Figure 2. Due to the imbalanced ratio of non-stroke to stroke patients in this work, the SMOTENC balancing technique was applied to training dataset before establishing the model. After normalization of data was completed, we applied KNN, SVM, MLP, LR, DT, RF, and XGBoost machine learning algorithms to train with the training dataset, and to test models with testing dataset and the independent testing dataset. The ROC curves of all seven models applied to the testing dataset and the independent testing dataset are given in Figure 3. The mean AUC values of 7 models in the validation cohort were 0.69, 0.76, 0.74, 0.75, 0.59, 0.78, and 0.78, respectively. Take the ROC curves into consideration, the XGB model performed best, with higher accuracy, sensitivity, specificity, and AUC values, they are 0.68 (0.57–0.78), 0.77 (0.63–0.9), 0.67 (0.53–0.8), 0.78 (0.75–0.81), respectively (Table 2). Due to the differences between the populations included in the database, the proportion of stroke patients and data characteristics of the independent testing cohort were distinct from those of the training set and validation set. Not surprisingly, we found the XGB model performed best in the independent testing set (Table 2). The accuracy, sensitivity, specificity, and AUC values of XGB model are 0.87 (0.78–0.93), 0.97 (0.96–0.98), 0.3 (0.19–0.45), 0.83 (0.79–0.87) respectively.

**TABLE 2:** Performance of machine learning methods in different data sets.

		Accuracy	Sensitivity	Specificity	AUC
The validating set	KNN	00.59 (0.47–0.65)	0.75 (0.65–0.9)	0.57 (0.43–0.64)	0.69 (0.66–0,73)
	LR	0.68 (0.55–0.79)	0.71 (0.55–0.86)	0.67 (0.51–0.82)	0.75 (0.71–0.78)
	RF	0.69 (0.56–0.79)	0.74 (0.6–0.88)	0.69 (0.53–0.81)	0.78 (0.74–0.81)
	DT	00.79 (0.77–0.81)	0.34 (0.26–0.41)	0.84 (0.82–0.87)	0.59 (0.55–0.63)
	SVM	0.69 (0.59–0.78)	0.75 (0.62–0.86)	0.68 (0.56–0.8)	0.76 (0.73–0.8)
	MLP	0.64 (0.52–0.76)	0.75 (0.58–0.89)	0.63 (0.47–0.78)	0.74 (0.7–0.77)
	XGB	0.68 (0.57–0.78)	0.77 (0.63–0.9)	0.67 (0.53–0.8)	0.78 (0.75–0.81)
The independent testing set	KNN	0.82 (0.72–0.87)	0.98 (0.97–0.99)	0.25 (0.16–0.31)	0.84 (0.81–0.88)
	LR	0.81 (0.68–0.9)	0.95 (0.94–0.96)	0.13 (0.1–0.2)	0.67 (0.65–0.69)
	RF	0.88 (0.79–0.93)	0.97 (0.96–0.98)	0.33 (0.2–0.49)	0.84 (0.8–0.87)
	DT	0.87 (0.84–0.89)	0.94 (0.94–0.95)	0.15 (0.09–0.24)	0.57 (0.51–0.63)
	SVM	0.87 (0.78–0.92)	0.96 (0.95–0.97)	0.26 (0.17–0.38)	0.77 (0.74–0.81)
	MLP	0.84 (0.75–0.91)	0.97 (0.96–0.98)	0.24 (0.16–0.35)	0.8 (0.76–0.84)
	XGB	0.87 (0.78–0.93)	0.97 (0.96–0.98)	0.3 (0.19–0.45)	0.83 (0.79–0.87)

**FIGURE 4:**
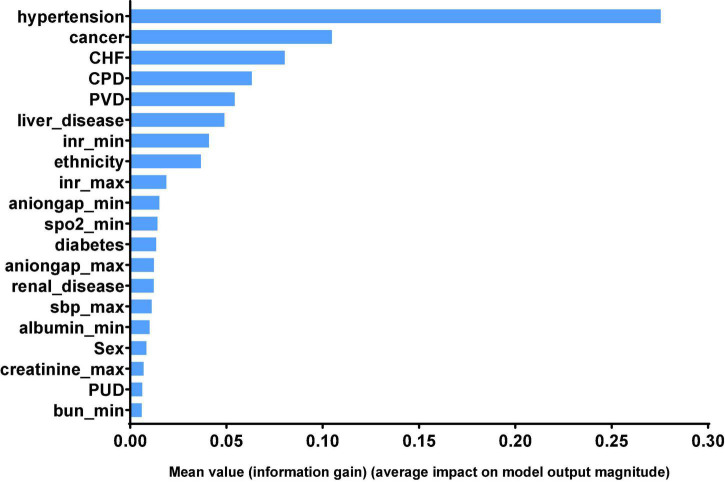
Significance of the predictors in the XGB model. The 20 variables with the highest relative importance are measured by the amount the variable reduced the information gain. CHF, congestive heart failure; CPD, chronic pulmonary disease; PVD, peripheral vascular disease; inr, international normalized ratio; spo2, Finger pulse oxygen saturation; sbp, systolic blood pressure; PUD, chronic pulmonary disease; bun, blood urea nitrogen.

Finally, we can get model-based importance of features, we present the importance of the predictors in the XGB model in Figure 4. The top five predictors were hypertension, cancer, congestive heart failure, chronic pulmonary disease and peripheral vascular disease (with importance values of 0.275, 0.104, 0.080, 0.063, and 0.054, respectively). The confusion matrix of the XGB model is presented in Table 3, which represents the predicted values vs. actual values for the validating and independent testing cohorts.

**TABLE 3:** Confusion matrix of the XGBoost model.

		Predicted: non-stroke	Predicted: stroke
The validating set	Actual: non-stroke	988	292
	Actual: stroke	52	94
The independent testing set	Actual: non-stroke	572	46
	Actual: stroke	19	24

## Discussion

In this study, we aimed to establish suitable model to recognize the possible stroke in elderly patients undergoing surgery and characterize the critical predictors of post-operative stroke. Nowadays, machine learning has been widely used in establishing disease prediction model.

With the help of ML methods, we found that the incidence of stroke in elderly patients undergoing surgery was associated with various clinical features. The XGB model performed best among the KNN, SVM, MLP, LR, DT, RF, and XGB models in our study. We identified hypertension, cancer, congestive heart failure, chronic pulmonary disease and peripheral vascular disease as predictors that were most associated with stroke.

Similar to our study, a study conducted using data from the Chinese Longitudinal Healthy Longevity Study built a stroke prediction model in elderly patients aged more than 60 years (Wu and Fang, 2020). It used SMOTH to deal with imbalanced data and selected important predictors as inputs in three machine learning methods. However, due to the different sources of patients and models, they found that sex, LDLC (low-density lipoprotein cholesterol), GLU (blood glucose), hypertension, and UA (uric acid) were the top five predictors in their RF model.

Compared with other studies, ours have certain strengths. This is the first study to establish stroke prediction models focused on elderly patients undergoing surgery by using an advanced machine learning method. We used several different methods to impute data (KNN, SoftImpute, IterativeImputer, IterativeSVD) and deal with imbalanced data (SMOTENC, ADASYN, BorderlineSMOTE, KMeansSMOTE, SVMSMOTE). Finally, we chose IterativeSVD and SMOTENC according to the AUC values. We utilized not only subjects from MIMIC-III for internal validation but also samples from the MIMIC-VI database for further external testing of the seven machine learning models.

Our study also has some limitations. First, relying on the results, we can only prevent stoke as much as possible, but cannot identify the stroke. The physiological signals like EMG, EEG, and ECG based monitoring system may have a chance to early identify stroke, which is also helpful to post-stroke treatment (Robinson et al., 2003; Hussain and Park, 2020, 2021a,b). Second, there were a certain number of missing values. We abandoned some potential confounding variables for having too many missing data, which is unavoidable in retrospective studies. Third, there were many variables involved, and the excessive variables increased the difficulty of model construction and the accuracy of the established models. Therefore further study about the effect of the strongest stroke predictors that we screened out should be carried out in the future.

## Conclusion

Our results showed that hypertension, cancer, congestive heart failure, chronic pulmonary disease and peripheral vascular disease might be closely associated with stroke in SICU elderly patients. The XGBoost model performs better than the KNN, SVM, MLP, LR, DT, and RF models in our study. In order to prevent stroke of elderly patients in SICU, we need to pay attention to their comorbidities more than other laboratory features, especially maintaining stable blood pressure. However, further additional verifications are necessary to examine the generalization of our models and predictors.

## Data Availability Statement

Publicly available datasets were analyzed in this study. This data can be found here: https://mimic.mit.edu/.

## Ethics Statement

Medical Information Mart for Intensive Care (MIMIC) is a large, freely-available medical database consisting of deidentified data from patients who were admitted to the critical care units of the Beth Israel Deaconess Medical Center. The consent was obtained at the beginning of data collection. Therefore, the ethical approval statement and the need for informed consent were jumped in this manuscript, which were not required for this study in accordance with the national legislation and the institutional requirements.

## Author Contributions

ZF, QW, and XZ made contributions to the conception and design of the work. XZ extracted the data from the MIMIC-III and MIMIC-VI databases. NF and XXZ participated in processing the data and the statistical analysis. XZ and NF finished the first draft, they contributed equally to this work and shared first authorship. All authors had revised the manuscript and approved the final edition.

## Conflict of Interest

The authors declare that the research was conducted in the absence of any commercial or financial relationships that could be construed as a potential conflict of interest.

## Publisher’s Note

All claims expressed in this article are solely those of the authors and do not necessarily represent those of their affiliated organizations, or those of the publisher, the editors and the reviewers. Any product that may be evaluated in this article, or claim that may be made by its manufacturer, is not guaranteed or endorsed by the publisher.
