# Supplementary material for: Machine Learning Prediction Models for Postoperative Stroke in Elderly Patients: Analyses of the MIMIC Database
Source: Front Aging Neurosci. 2022 Jul 18;14:897611. doi: 10.3389/fnagi.2022.897611 (PMC9341133; doi:10.3389/fnagi.2022.897611)
Supplement: Supplementary file 1 [file Data_Sheet_1.pdf]

Supplementary Material

Supplementary Data

Supplementary Table 1. Demographic and clinical characteristics of training and validation cohorts.

Variables	Total (n = 7128)	Training set (n = 5702)	Validation set (n = 1426)	p
**Demographic characteristics**				
**age, Mean ± SD**	72.3 ± 10.4	72.3 ± 10.4	72.3 ± 10.4	0.814
**Gender, Female n (%)**	3426 (48.1)	2723 (47.8)	703 (49.3)	0.300
**Race, n (%)**				0.218
**ASIAN, n (%)**	222 (3.1)	183 (3.2)	39 (2.7)	0.402
**BLACK, n (%)**	592 (8.3)	458 (8)	134 (9.4)	0.106
**WHITE, n (%)**	5083 (71.3)	4062 (71.2)	1021 (71.6)	0.813
**OTHER, n (%)**	1231 (17.3)	999 (17.5)	232 (16.3)	0.281
**BMI, Mean ± SD**	1300.8 ± 85503.1	1585.1 ± 95587.0	163.9 ± 2934.9	0.575
**Comorbidities**				
**CHF, n (%)**	1483 (20.8)	1184 (20.8)	299 (21)	0.895
**PVD, n (%)**	927 (13.0)	737 (12.9)	190 (13.3)	0.722
**hypertension, n (%)**	2794 (39.2)	2247 (39.4)	547 (38.4)	0.487
**CPD, n (%)**	1712 (24.0)	1370 (24)	342 (24)	1
**diabetes, n (%)**	2070 (29.0)	1636 (28.7)	434 (30.4)	0.206
**renal_disease, n (%)**	1349 (18.9)	1075 (18.9)	274 (19.2)	0.784
**liver_disease, n (%)**	844 (11.8)	681 (11.9)	163 (11.4)	0.624
**PUD, n (%)**	204 (2.9)	165 (2.9)	39 (2.7)	0.816
**cancer, n (%)**	1405 (19.7)	1101 (19.3)	304 (21.3)	0.095
**rheumatic_disease, n (%)**	259 (3.6)	207 (3.6)	52 (3.6)	1
**sepsis, n (%)**	3033 (42.6)	2452 (43)	581 (40.7)	0.13
**Laboratory results**				
**spo2_min, Mean ± SD**	91.8 ± 7.1	91.8 ± 7.1	91.9 ± 6.9	0.886
**spo2_mean, Mean ± SD**	96.8 ± 2.6	96.8 ± 2.6	96.8 ± 2.8	0.794
**aniongap_min, Mean ± SD**	13.5 ± 3.3	13.5 ± 3.3	13.5 ± 3.3	0.465
**aniongap_max, Mean ± SD**	16.2 ± 4.3	16.2 ± 4.3	16.3 ± 4.4	0.785
**albumin_min, Mean ± SD**	3.3 ± 0.5	3.3 ± 0.5	3.3 ± 0.5	0.801
**albumin_max, Mean ± SD**	3.4 ± 0.5	3.4 ± 0.5	3.4 ± 0.5	0.754
**glucose_mean, Mean ± SD**	140.5 ± 49.0	140.8 ± 48.8	139.6 ± 49.8	0.418
**potassium_min, Mean ± SD**	3.8 ± 0.4	3.8 ± 0.4	3.8 ± 0.4	0.578
**potassium_max, Mean ± SD**	4.1 ± 0.5	4.1 ± 0.5	4.1 ± 0.5	0.961
**bilirubin_total_min, Median (IQR)**	0.8 (0.4, 1.6)	0.8 (0.4, 1.5)	0.8 (0.4, 1.6)	0.556
**bilirubin_total_max, Median (IQR)**	1.0 (0.4, 1.9)	1.0 (0.4, 1.9)	1.0 (0.4, 2.0)	0.639
**creatinine_min, Median (IQR)**	0.9 (0.7, 1.2)	0.9 (0.7, 1.2)	0.9 (0.7, 1.2)	0.793
**creatinine_max, Median (IQR)**	1.0 (0.8, 1.4)	1.0 (0.8, 1.4)	1.0 (0.8, 1.5)	0.498
**lactate_min, Median (IQR)**	1.5 (1.2, 1.9)	1.5 (1.2, 1.9)	1.5 (1.2, 1.8)	0.496

**lactate_max, Median (IQR)**	2.0 (1.4, 2.9)	2.0 (1.4, 2.9)	2.0 (1.4, 2.8)	0.317
**platelets_min, Median (IQR)**	193.0 (141.0, 253.0)	193.0 (141.0, 253.0)	194.0 (144.0, 253.0)	0.547
**platelets_max, Median (IQR)**	218.0 (165.0, 285.0)	218.0 (164.2, 285.0)	219.0 (165.0, 284.0)	0.654
**ptt_min, Median (IQR)**	28.3 (25.0, 32.6)	28.3 (25.0, 32.6)	28.4 (25.1, 32.8)	0.43
**ptt_max, Median (IQR)**	30.5 (26.5, 40.2)	30.5 (26.5, 40.0)	30.5 (26.5, 41.9)	0.557
**inr_min, Median (IQR)**	1.2 (1.1, 1.4)	1.2 (1.1, 1.4)	1.2 (1.0, 1.4)	0.356
**inr_max, Median (IQR)**	1.2 (1.1, 1.6)	1.2 (1.1, 1.6)	1.2 (1.1, 1.7)	0.832
**pt_min, Median (IQR)**	13.0 (11.7, 15.1)	13.0 (11.7, 15.1)	13.0 (11.7, 15.3)	0.302
**pt_max, Median (IQR)**	13.8 (12.1, 18.8)	13.8 (12.1, 18.7)	13.7 (12.1, 19.8)	0.935
**bun_min, Median (IQR)**	18.0 (12.0, 26.2)	18.0 (13.0, 27.0)	17.0 (12.0, 26.0)	0.981
**bun_max, Median (IQR)**	20.0 (15.0, 31.0)	20.0 (15.0, 31.0)	21.0 (14.0, 31.0)	0.903
**wbc_min, Median (IQR)**	9.3 (6.8, 12.6)	9.3 (6.8, 12.6)	9.2 (6.9, 12.6)	0.663
**wbc_max, Median (IQR)**	11.7 (8.6, 15.9)	11.7 (8.6, 15.9)	11.7 (8.7, 16.2)	0.803
**Vital signs**				
**TP_mean, Mean ± SD**	100.8 ± 20.3	101.0 ± 20.3	100.2 ± 20.5	0.201
**HR_max, Mean ± SD**	82.4 ± 15.0	82.4 ± 15.0	82.2 ± 15.2	0.694
**HR_mean, Mean ± SD**	153.9 ± 23.3	154.2 ± 23.3	153.0 ± 23.3	0.09
**sbp_max, Mean ± SD**	123.5 ± 17.4	123.6 ± 17.3	123.3 ± 17.5	0.513
**sbp_mean, Mean ± SD**	88.0 ± 20.2	88.2 ± 20.1	87.6 ± 20.4	0.342
**dbp_max, Mean ± SD**	62.6 ± 10.7	62.6 ± 10.6	62.4 ± 11.1	0.571
**dbp_mean, Mean ± SD**	106.6 ± 24.0	106.8 ± 23.9	105.8 ± 24.4	0.193
**mbp_max, Mean ± SD**	79.5 ± 11.0	79.6 ± 10.9	79.3 ± 11.5	0.298
**mbp_mean, Mean ± SD**	36.9 ± 0.6	36.9 ± 0.6	36.9 ± 0.6	0.532

Supplementary Table 2. Demographic and clinical characteristics of independent cohorts for stroke group and non-stroke group across all subjects.

Variables	Total (n = 661)	Non-stroke (n = 618)	Stroke (n = 43)	*p*-value
**Demographic characteristics**				
**age, Mean ± SD**	70.7 ± 10.0	70.6 ± 10.0	71.2 ± 9.8	0.724
**Gender, Female n (%)**	262 (39.6)	245 (39.6)	17 (39.5)	0.9887
**Race, n (%)**				0.014
**Asian**	20 (3.0)	17 (2.8)	3 (7)	
**Black**	58 (8.8)	55 (8.9)	3 (7)	
**White**	92 (13.9)	80 (12.9)	12 (27.9)	
**Others**	491 (74.3)	466 (75.4)	25 (58.1)	
**BMI, Mean ± SD**	28.2 ± 6.7	28.3 ± 6.6	27.2 ± 7.4	0.3
**Comorbidities**				
**CHF, n (%)**	152 (23.0)	144 (23.3)	8 (18.6)	0.603
**PVD, n (%)**	76 (11.5)	72 (11.7)	4 (9.3)	0.807
**hypertension, n (%)**	91 (13.8)	86 (13.9)	5 (11.6)	0.848
**CPD, n (%)**	125 (18.9)	113 (18.3)	12 (27.9)	0.175
**diabetes, n (%)**	204 (30.9)	188 (30.4)	16 (37.2)	0.447
**renal_disease, n (%)**	123 (18.6)	118 (19.1)	5 (11.6)	0.311
**liver_disease, n (%)**	142 (21.5)	135 (21.8)	7 (16.3)	0.505

**peptic_ulcer_disease, n (%)**	1 (0.2)	1 (0.2)	0 (0)	1
**cancer, n (%)**	104 (15.7)	103 (16.7)	1 (2.3)	0.023
**rheumatic_disease, n (%)**	24 (3.6)	24 (3.9)	0 (0)	0.393
**sepsis, n (%)**	134 (20.3)	132 (21.4)	2 (4.7)	0.015
**Laboratory results**				
**spo2_min, Mean ± SD**	90.8 ± 11.4	90.7 ± 11.2	91.5 ± 14.4	0.642
**spo2_mean, Mean ± SD**	97.4 ± 3.0	97.4 ± 3.0	98.0 ± 2.0	0.174
**aniongap_min, Mean ± SD**	13.2 ± 3.8	13.2 ± 3.8	13.4 ± 2.9	0.676
**aniongap_max, Mean ± SD**	17.4 ± 5.3	17.5 ± 5.4	16.8 ± 3.5	0.413
**albumin_min, Mean ± SD**	2.8 ± 0.7	2.8 ± 0.7	3.4 ± 0.8	< 0.001
**albumin_max, Mean ± SD**	3.0 ± 0.7	2.9 ± 0.7	3.5 ± 0.7	< 0.001
**glucose_mean, Mean ± SD**	145.3 ± 42.3	144.7 ± 42.6	153.5 ± 37.7	0.188
**potassium_min, Mean ± SD**	3.7 ± 0.6	3.7 ± 0.6	3.6 ± 0.5	0.251
**potassium_max, Mean ± SD**	4.7 ± 0.8	4.7 ± 0.8	4.4 ± 0.6	0.012
**bilirubin_total_min, Median (IQR)**	0.8 (0.4, 1.8)	0.8 (0.4, 1.9)	0.6 (0.4, 1.1)	0.07
**bilirubin_total_max, Median (IQR)**	1.0 (0.5, 2.7)	1.1 (0.5, 2.8)	0.7 (0.4, 1.2)	0.019
**creatinine_min, Median (IQR)**	1.1 (0.8, 1.8)	1.1 (0.8, 1.8)	0.9 (0.7, 1.4)	0.07
**creatinine_max, Median (IQR)**	1.4 (1.0, 2.3)	1.4 (1.0, 2.3)	1.1 (0.9, 1.6)	0.06
**lactate_max, Median (IQR)**	1.6 (1.1, 2.2)	1.6 (1.1, 2.2)	1.7 (1.2, 2.4)	0.537
**lactate_min, Median (IQR)**	2.8 (1.7, 5.3)	2.9 (1.7, 5.4)	2.2 (1.4, 3.6)	0.018
**platelets_min, Median (IQR)**	166.0 (95.0, 245.0)	161.0 (90.0, 244.8)	190.0 (135.5, 253.5)	0.028
**platelets_max, Median (IQR)**	214.0 (145.0, 315.0)	210.5 (144.0, 314.8)	225.0 (189.5, 312.5)	0.2
**ptt_min, Median (IQR)**	30.0 (26.0, 33.9)	30.1 (26.2, 34.1)	25.6 (23.3, 29.9)	< 0.001
**ptt_max, Median (IQR)**	36.9 (29.3, 57.5)	37.3 (29.6, 58.1)	30.1 (25.8, 42.4)	0.002
**inr_min, Median (IQR)**	1.3 (1.1, 1.5)	1.3 (1.1, 1.5)	1.1 (1.0, 1.3)	< 0.001
**inr_max, Median (IQR)**	1.5 (1.2, 2.0)	1.5 (1.2, 2.0)	1.3 (1.1, 1.8)	0.003
**pt_min, Median (IQR)**	14.2 (13.0, 15.9)	14.2 (13.1, 16.1)	13.1 (11.9, 14.5)	< 0.001
**pt_max, Median (IQR)**	16.0 (13.9, 19.9)	16.1 (13.9, 19.9)	14.2 (12.6, 18.1)	0.003
**bun_min, Median (IQR)**	22.0 (15.0, 38.0)	23.0 (15.0, 38.0)	15.0 (13.0, 25.5)	0.003
**bun_max, Median (IQR)**	28.0 (19.0, 44.0)	29.0 (19.0, 44.0)	19.0 (15.5, 28.5)	< 0.001
**wbc_min, Median (IQR)**	9.4 (6.1, 13.1)	9.4 (6.0, 13.0)	10.1 (7.0, 13.8)	0.174
**wbc_max, Median (IQR)**	13.7 (9.7, 18.8)	13.7 (9.7, 18.9)	14.3 (11.8, 16.8)	0.633
**Vital signs**				
**TP_mean, Mean ± SD**	106.9 ± 22.7	106.9 ± 22.7	106.6 ± 23.7	0.915
**HR_max, Mean ± SD**	87.0 ± 16.2	87.1 ± 16.2	84.5 ± 15.3	0.301
**HR, Mean ± SD**	156.1 ± 24.4	155.5 ± 24.3	164.9 ± 23.8	0.014
**sbp_max, Mean ± SD**	120.1 ± 17.6	119.4 ± 17.4	130.4 ± 16.7	< 0.001
**sbp_mean, Mean ± SD**	82.7 ± 19.9	82.1 ± 19.7	92.2 ± 20.8	0.001
**dbp_max, Mean ± SD**	58.8 ± 10.8	58.5 ± 10.6	63.8 ± 12.4	0.002
**dbp_mean, Mean ± SD**	108.5 ± 27.6	108.3 ± 28.0	111.3 ± 22.1	0.487
**mbp_max, Mean ± SD**	77.8 ± 11.6	77.4 ± 11.6	82.7 ± 11.6	0.004
**mbp_mean, Mean ± SD**	36.9 ± 0.7	36.9 ± 0.7	37.1 ± 0.6	0.033
